# Bounded Asymmetry in Road Networks

**DOI:** 10.1038/s41598-019-48463-z

**Published:** 2019-08-16

**Authors:** Juan C. Martínez Mori, Samitha Samaranayake

**Affiliations:** 1 Center for Applied Mathematics, Cornell University, Ithaca, NY 14853 USA; 2 School of Civil and Environmental Engineering, Cornell University, Ithaca, NY 14853 USA

**Keywords:** Civil engineering, Applied mathematics, Computer science, Scientific data

## Abstract

Road networks are a classical stage for applications in network science and graph theory. Meanwhile, many combinatorial problems that arise in road networks are computationally intractable. Thus, an attractive way of tackling them is through efficient heuristics with provable performance guarantees, better known as approximation algorithms. This motivates the intersection of algorithm design with the aforementioned fields. Specifically, identifying measures that characterize graphs and exploiting them in the design of algorithms may yield practical heuristics with rigorous mathematical justification. Herein, we propose a new graph measure, namely the asymmetry factor Δ_*G*_ of a directed graph *G*, with immediate algorithmic results via a symmetrization procedure and the black box use of approximation algorithms for symmetric graphs. Crucially, we analyze the asymmetry factors of the road networks from a diverse set of twelve cities, providing empirical evidence that road networks exhibit low bounded asymmetry and thereby justifying the practical use of algorithms for symmetric graphs.

## Introduction

Road networks are one of the classical stageas for applications in network science and graph theory. The reason for this is that they fundamentally shape the exchange of goods, services, and information that are indispensable for daily life since the dawn of human existence. More broadly, road networks in cities and regions are testaments and enablers of cultural, economic, aesthetic, and political values^1–4^. For example, ancient Romans built the *viae Romanae*; the road network interconnecting and hence empowering the Roman Empire, along with a sophisticated system of road-side milestones and published maps and *itineraria*, which specified the ‘shortest’ paths between pairs of cities^4,5^. The objective was to provide useful guidance to traders and other travelers, and it is parallel to modern navigation tools like Google Maps^TM^ and Waze^TM^. Before becoming the sixteenth U.S. President, Abraham Lincoln served in the Eighth Judicial Circuit, touring fourteen counties in the State of Illinois every Fall and Spring^6^. Although Lincoln’s tour was not the shortest possible, there is clear evidence suggesting that intent was to minimize the travel of court members^6^. In short, many elementary transportation problems arising in daily life are naturally ingrained in road networks.

However, it was rather recently that we started studying road networks with mathematical formality. In 1735, Euler resolved the long-standing Bridges of Königsberg Problem, which asked whether it was possible to perform a tour through the town while visiting each bridge in a set of seven exactly once^7^. He was able to solve the problem by viewing it through the lens of an abstraction; namely a set of points connected by edges representing bridges. This abstraction, which we refer to as a graph, laid the foundations of modern graph theory. A century later, Hamilton studied the problem of finding a tour on a graph, except now it was the points that could be visited only once^6^. Interestingly, given an arbitrary graph, determining whether a Hamiltonian tour could be performed appeared to be much harder than doing the same for an Eulerian tour. This suspicion was not demystified until the 1970’s, when Karp^8^ elaborated on theory by Cook^9^ to show that the Hamiltonian Tour Problem belongs to a class of seemingly distinct problems for which no efficient algorithms are known. The problems belonging to this class are referred to as *NP*-complete, and they equivalent in the sense that, if there exists an efficient algorithm to solve one of them, there exist efficient algorithms to solve each and every one of them. The optimization versions of these problems are referred to as *NP*-hard. To this date, it remains an open question whether such an algorithm exists: this is the renowned *P* versus *NP* millennium question^10^, where *P* and *NP* are the classes of decision problems that can be solved and verified in polynomial time, respectively. For instance, Lincoln’s circuit tour problem is more popularly known as the Traveling Salesman Problem (TSP), whose decision version is *NP*-complete.

Despite this fact, *NP*-hard transportation problems in the real world are still being ‘solved’ on a regular basis. By solved we mean that we use efficient algorithms that may not always find the absolute best solution, but that for most practical purposes produce feasible solutions of reasonable quality. These are referred to as heuristics, and they are of interest because unless *P* = *NP*, there does not exist an algorithm that solves an *NP*-complete problem efficiently. One important class of heuristics are known as approximation algorithms, namely those that have provable theoretical guarantees regarding the quality of the solution. To be precise, a polynomial time algorithm for a minimization problem is said to be an *α*-approximation algorithm if, for all instances of the problem, it produces a solution whose value is within an *α* factor of the value of the optimal solution.

Typically, approximation algorithms are designed around the idea of rigorously identifying and exploiting structure in the problem of interest. This strategy is in fact not limited to approximation algorithms. For example, there exists extensive algorithm engineering literature proposing graph measures that formally address the question of why route planning speed-up heuristics work in practice, such as the highway dimension^5^ and the skeleton dimension^11^. Bast *et al*.^12^ provide an excellent survey on practical algorithms for route planning. Meanwhile, there has been a growing interest by network scientists and urbanists in characterizing road networks and other abstractions of the urban environment. One line of work has dealt with defining distance measures for road network comparison^13–15^, the motivation being assessing the quality of networks reconstructed from vehicle trajectory data and identifying network changes over time. A different line of work has proposed a variety of network measures, including node connectivity and path lengths^16^, node and edge centrality^17^, block and intersection geometry^18,19^, graph circuity^20^, and graph planarity^21^. Boeing^22^ proposes a comprehensive typology for many of the measures above in the context of road networks.

From a practical point of view, a particularly interesting direction of research is in the intersection of algorithm design and network science. Specifically, identifying measures that characterize road networks and exploiting them in the design of algorithms has a lot of potential to produce practical heuristics with rigorous mathematical justification. For instance, the approximate planarity of road networks gives practical value to the work of Gharan and Saberi^23^; an approximation algorithm for the Asymmetric Traveling Salesman Problem (ATSP) on graphs with bounded genus. Their elegant theory is of independent interest. The same can be said regarding the low Highway-Dimension of some road networks and the work of Feldmann *et al*.^24^.

In this paper, we propose a new graph measure that has immediate algorithmic implications. Namely, we consider the asymmetry factor Δ_*G*_ of a directed graph *G*, which is the maximum ratio between the lengths of shortest paths from *u* to *v* and *v* to *u* for any pair of distinct nodes *u* and *v* in *G*. We show that directed graphs with bounded asymmetry Δ_*G*_ allow practical constant factor approximation algorithms for some discrete optimization problems such as, but not restricted to, the ATSP via a symmetrization procedure and the black box use of constant factor approximation algorithms for symmetric graphs. This result is especially beneficial when Δ_*G*_ is a small constant, as algorithms for symmetric problems tend to enjoy better theoretical guarantees and ease of implementation. Crucially, we analyze the asymmetry factors of the road networks from a diverse set of twelve cities around the world, providing empirical evidence that road networks do in fact exhibit bounded asymmetry. Moreover, we dissect the raw evidence to argue that the asymmetry quickly becomes especially low as we restrict ourselves to only consider nodes with increasingly large distance between them. This is of practical interest, as many applications are mainly concerned with nodes of non-negligible distance in between. We are able to do this through the Python library OSMNX, which is an extensive library developed by Boeing^25^ for analyzing urban road networks.

The remainder of this paper is organized as follows. In the next section we describe the mathematical model representing a road network and outline our experimental methods. Then, we describe the immediate implications of directed graphs with bounded asymmetry in the context of combinatorial optimization. We then present our empirical results regarding the asymmetry of road networks for a diverse set of cities around the world. Lastly, we discuss our empirical results and their practical applications.

## Materials and Methods

### Mathematical model

We adopt the following mathematical model. Let *R* = (*V*,*E*_*r*_,*l*_*r*_) be a weighted strongly-connected graph representing a road network. The nodes *v* ∈ *V* correspond to road intersections and the edges *e* = (*u*,*v*) ∈ *E*_*r*_ correspond to directed road segments. The weight *l*_*r*_(*u*,*v*) > 0 of an edge (*u*,*v*) ∈ *E*_*r*_ is equal to its length in meters. Given *R*, we prepare a complete directed graph *G* = (*V*,*E*,*l*) on the same node set *V*, where we let *l*(*u*,*v*) be the length of the shortest path from *u* to *v* in *R*. In other words, in *G* we extend the interpretation of an edge (*u*,*v*) ∈ *E* from that of a single directed road segment to that of a sequence of directed road segments that collectively compose a shortest path from *u* to *v*. This technique is known as the metric closure of *R* and can be performed in polynomial time. Moreover, *G* satisfies the triangle inequality *l*(*u*,*v*) ≤ *l*(*u*,*w*) + *l*(*w*,*v*) for all *u*,*w*,*v* ∈ *V*. Note, however, that *l*(*u*,*v*) ≠ *l*(*v*,*u*) in general for *u*,*v* ∈ *V*. Therefore, we define the asymmetry factor of a pair of nodes *u* ≠ *v* ∈ *V* as Δ_{*u*,*v*}_ = max{*l*(*u*,*v*)/*l*(*v*,*u*),*l*(*v*,*u*)/*l*(*u*,*v*)}. Lastly, let Δ_*G*_ = max_*u*_ ≠ _*v*_ ∈ _*V*_{Δ_{*u*,*v*}_} be the maximum asymmetry factor between any pair of nodes *u* ≠ *v* ∈ *V*. We refer to Δ_*G*_ as the asymmetry of *G*.

Note that since *R* always represents a road network with strictly positive segment lengths, it holds that Δ_*G*_ is always bounded by some possibly large constant specific to the city or region at hand. Thus, a more interesting question is how large Δ_*G*_ can be. This is the central topic of our empirical results.

### Experimental methods

**Figure 1:**
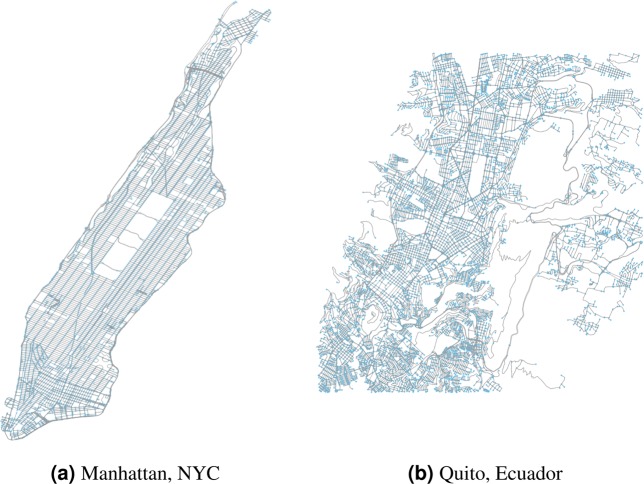
Examples of road networks *R* obtained via OSMNX^25,26^.

In this work, we investigate the pairwise asymmetries of road networks from a diverse set of cities. In particular, we are interested in establishing empirical bounds on Δ_*G*_, facilitating the algorithmic results outlined above. All computations are done using Python 3.6. For each city under study, we obtain a graph *R* and subsequently prepare *G* in accord to the mathematical model described above using the Python library OSMNX^25^, version 0.8.2, which queries the drivable road network from Open Street Map^26^. Examples of such a networks are displayed in Fig. 1. Finally, we compute the asymmetry factor of every pair of nodes *u* ≠ *v* ∈ *V*.

The precise bounding box coordinates for each query can be found in the Supplementary Material. In each case, the bounding box is intended to pivot at the city centre. Recalling that the metric closure of a graph of *n* nodes has *O*(*n*^2^) edges, the size of the box is intended to cover an area as large as possible while inducing a metric closure that consumes around 12 GB of memory. The metric closures *G* are prepared by computing the shortest paths between all pairs of nodes in each city. This is done using the Python library NetworkX^27^, version 2.2, which implements shortest path algorithms. The asymmetry factors are obtained trivially. All figures are prepared using open-source Python libraries^28,29^. The open-source nature of the data sets and libraries used makes this research readily reproducible for essentially any city or region supported by Open Street Map. The source code developed to obtain the results presented will be provided through a publicly available repository.

## Results

### Implications of bounded asymmetry

We now discuss some immediate implications of bounded asymmetry in graphs. We focus on minimization problems, but the results carry over to maximization problems with appropriate changes to the definition of approximation algorithms. Suppose we are given a complete directed graph *G* = (*V*,*E*,*l*) satisfying the triangle inequality, such as the graph described above. This graph may be for instance the metric closure of some underlying graph. Moreover, assume that Δ_*G*_ is bounded. Consider the following symmetrization procedure: for all *u*,*v* ∈ *V*, replace *l*(*u*,*v*) with *l*′(*u*,*v*) = max{*l*(*u*,*v*),*l*(*v*,*u*)}.

First, note that the procedure can be trivially performed in polynomial time. Second, note that the procedure ensures that *l*′(*u*,*v*) ≤ Δ_*G*_⋅*l*(*u*,*v*) for any *u*,*v* ∈ *V*. Hence the total length of any subset of edges (e.g., a tour, a path, a tree) increases at most by a factor of Δ_*G*_. Third, note that the procedure preserves the triangle inequality; i.e., *l*′(*u*,*v*) ≤ *l*′(*u*,*w*) + *l*′(*w*,*v*) for any *u*,*w*,*v* ∈ *V*. This can be shown as follows. By the triangle inequality (of the original graph), we have that *l*(*u*,*v*) ≤ *l*(*u*,*w*) + *l*(*w*,*v*). If *l*′(*u*,*v*) = *l*(*u*,*v*), the inequality is preserved because the right hand side of the inequality cannot decrease. If *l*′(*u*,*v*) = *l*(*v*,*u*) and *l*′(*u*,*w*) = *l*(*w*,*u*) we have that *l*′(*u*,*v*) = *l*(*v*,*u*) ≤ *l*(*v*,*w*) + *l*(*w*,*u*) = *l*(*v*,*w*) + *l*′(*u*,*w*) ≤ *l*′(*w*,*v*) + *l*′(*u*,*w*), where the first inequality follows from the triangle inequality (of the original graph) and the last inequality follows because *l*′(*w*,*v*) ≥ *l*(*v*,*w*). A nearly identical argument can be made if *l*′(*u*,*v*) = *l*(*v*,*u*) and *l*′(*w*,*v*) = *l*(*v*,*w*) or if *l*′(*u*,*v*) = *l*(*v*,*u*), *l*′(*u*,*w*) = *l*(*w*,*u*), and *l*′(*w*,*v*) = *l*(*v*,*w*).

Therefore, we are left with a complete symmetric graph *G*′ = (*V*,*E*,*l*′) satisfying the triangle inequality. In turn this means that, at the expense of a factor of Δ_*G*_ in the approximation guarantee, we are able to approximately and efficiently solve a variety of minimization problems defined on directed graphs via approximation algorithms for their undirected counterparts. More precisely, for a minimization problem on a complete directed graph *G* = (*V*,*E*,*l*) satisfying the triangle inequality and with asymmetry factor Δ_*G*_ such that the objective value is solely a function of the edge set *E* and its weight *l*, the symmetrization procedure together with any *α*-approximation algorithm for the problem’s symmetric counterpart will lead to a (*α*⋅Δ_*G*_)-approximation to the original problem. This result is highly desirable when Δ_*G*_ is small, as symmetric algorithms tend to exhibit more promising theoretical guarantees while enjoying simplicity and ease of implementation.

In the following discussion we focus on the Asymmetric Traveling Salesman Problem (ATSP) and the Directed Steiner Tree Problem (DTSP), and variants, as concrete examples for which a bounded asymmetry factor is advantageous. However, we emphasize that the implications of bounded asymmetry are not specific to these problems. For example, the results also hold for the *k*-server problem and its asymmetric counterpart^30^, for which no competitive algorithms exists^31^. Neither they are specific to any particular class of graphs. Indeed, the mathematical result holds for any graph with bounded asymmetry; our technical contribution is the empirical analysis of the asymmetry factors in the specific context of road networks.

In the ATSP, we are given a directed graph *G* and we are concerned with finding a tour of minimum cost that visits each node *v* ∈ *V* at least once. Unfortunately, the decision versions of the ATSP and its undirected counterpart, the metric Traveling Salesman Problem (TSP), are *NP*-complete via a reduction from the Hamiltonian Cycle Problem. In a recent theoretical breakthrough, Svensson, Tarnawski, and Végh presented the first constant factor approximation algorithm for the ATSP^32^, with a 5,500 approximation factor. More classical approximation algorithms are asymptotically worse as their approximation factors depend logarithmically on *n* = |*V*|, e.g., they are *O*(log *n*)^33,34^ and *O*(log *n*/log log *n*)^35^; we denote base two logarithms by log throughout. To put this in context, the Manhattan and USA road networks have nearly 4,500 and 24,000,000 nodes^36^ respectively, yielding log 4,500 ≈ 12 and log 24,000,000 ≈ 24.5. Gharan and Saberi^23^ developed a constant factor approximation algorithm for the ATSP on graphs with bounded genus, which is a concept in topological graph theory. For the special case of planar graphs, their algorithm has a 22.5(1 + 1/*n*) approximation factor. However, if we are able to establish that Δ_*G*_ is bounded, we may simply use Christofides’ algorithm^37^ for the metric Traveling Salesman Problem (TSP) on *G*′ to obtain a $(\frac{3}{2}\cdot {{\rm{\Delta }}}_{G})$-approximation. This is especially beneficial if Δ_*G*_ is a small constant. Similar results are immediately obtained for variants of the ATSP and their undirected counterparts, such as the Path-TSP^38,39^, the Prize-collecting TSP^40^, the Orienteering Problem^41^, and the Generalized TSP^42^.

In the DSTP, we are given a directed graph *G* and we are concerned with finding a minimum cost arborescence rooted at a pre-specified node *r* ∈ *V* that includes all the vertices *v* ∈ *X* for some *X* ⊆ *V* called the terminal nodes. The decision versions of the DTSP and its undirected counterpart, the Steiner Tree Problem (STP), are classic *NP*-complete problems via a reduction from the Exact Cover Problem^8^. Multiple constant factor approximation algorithms for the STP have been proposed^40,43,44^, the best of which achieves a 1.39-approximation^44^. Meanwhile, the only known approximation algorithms for the DTSP depend logarithmically on the number of nodes to visit *k* when allowed to run in quasi-polynomial time^45,46^. As before, if we are able to establish that Δ_*G*_ is bounded, we may simply use existing approximation algorithms for the undirected counterpart on *G*′ at the expense of a factor of Δ_*G*_ in the approximation guarantee. Again, similar results are immediately obtained for variants of the problem, such as the Group Steiner Tree Problem^40,42^.

### Empirical evidence of bounded asymmetry

In the interest of avoiding confirmation bias, we conform to a typology of street patterns that classifies urban areas into one of four categories based on their topological footprint due to Louf and Barthelemy^19^. This typology is particularly relevant because it is a global-scale typology that focuses not only on the adjacency matrix of the graph defined by the street network, but also on the spatial distribution of nodes and edges, which ultimately define the street network’s geometry. In turn, the typology may provide insight regarding the impact of the street network’s geometry on the asymmetry factors. Due to the size of our experiments, we study only a subset of 12 cities (out of the 131 cities considered in the typology^19^) spanning all four categories and all continents except Antartica. We study (*i*) Buenos Aires, Argentina from Category 1, (*ii*) Athens, Greece; Quito, Ecuador; Chennai, India; Vancouver, British Columbia (BC); and Tokyo, Japan from Category 2, (*iii*) New Orleans, Louisiana (LA); Manhattan, New York City (NYC); Barcelona, Spain; Moscow, Russia; and Auckland, New Zealand (NZ) from Category 3, and (*iv*) Mogadishu, Somalia from Category 4. Note that in the original paper proposing the typology, each of Category 1 and Category 4 contain a single city due to their lack of prevalence. We emphasize that since the experiments are highly reproducible, the validity of our results may be easily corroborated for any city of interest.

For conciseness, in this section we mainly focus on the representatives of each category of primary discussion in the original paper proposing the urban street pattern typology^19^, namely Buenos Aires, Argentina; Athens, Greece; New Orleans, LA; and Mogadishu, Somalia, together with Manhattan, NYC and Quito, Ecuador. The latter two were selected due to their prominence and unique geographic topology, respectively. The corresponding results for the remaining cities can be found in the Supplementary Material. Our observations for the remaining cities are the same as the ones outlined in this section.

**Figure 2:**
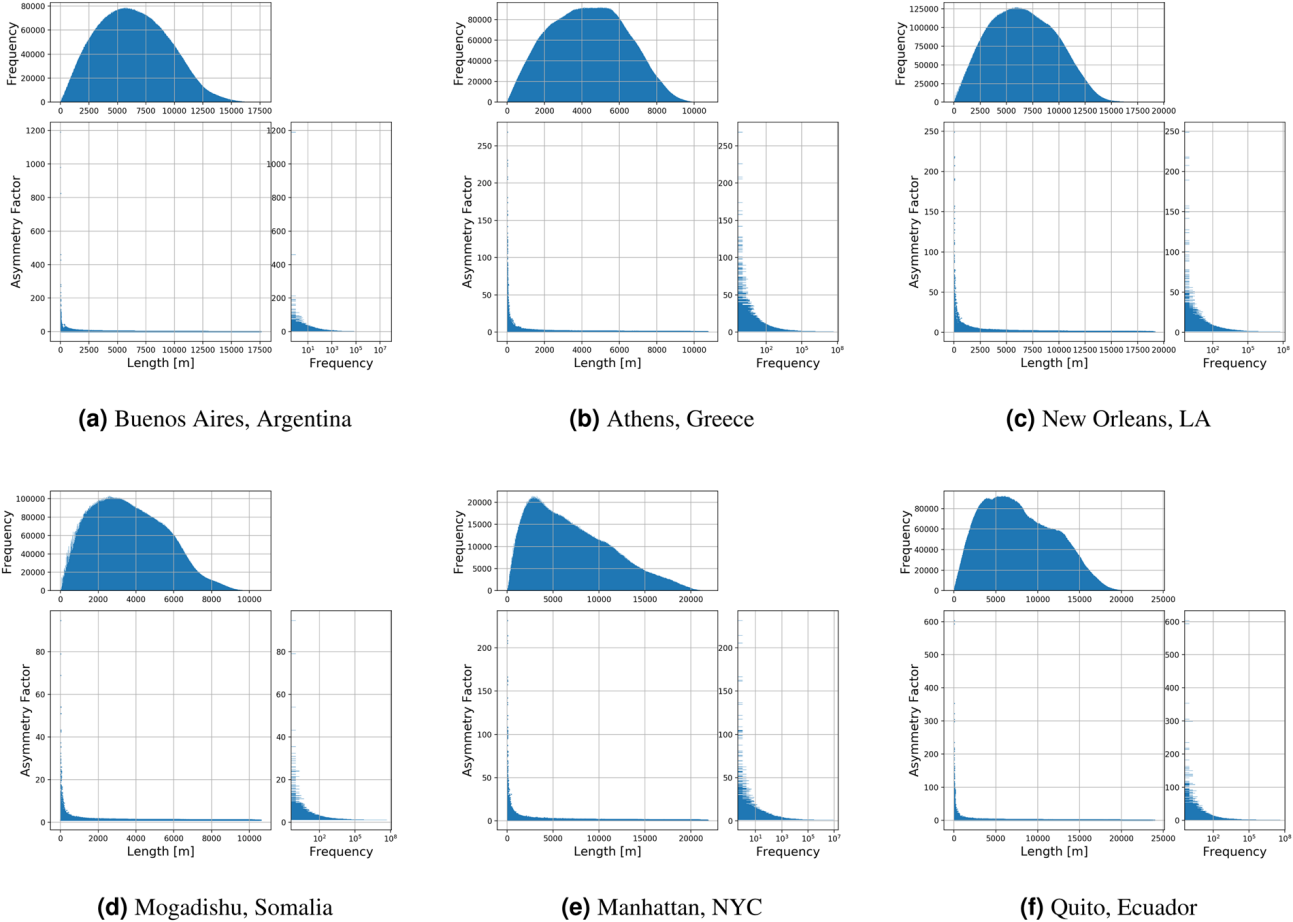
Scatter plots of asymmetry factors of all pairs of nodes *u* ≠ *v* ∈ *V* as a function of the length between them, namely min {*l*(*u*,*v*), *l*(*v*,*u*)}. The marginals frequencies of the lengths and asymmetry factors are included on the top and to the right of the scatter plot, respectively. The marginal frequencies of the asymmetry factors are on a logarithmic scale.

Figure 2 displays scatter plots of the asymmetry factors of all pairs of nodes *u* ≠ *v* ∈ *V* as a function of the length of the shortest of the edges between them, namely min{*l*(*u*,*v*), *l*(*v*,*u*)}, which always exist due to our construction of *G*. On the top and on the right of the scatter plots we include the marginal frequencies of the lengths and asymmetry factors, respectively. Note that the marginal frequencies of the asymmetry factors are on a logarithmic scale. Our main findings are twofold: (*i*) high asymmetries occur, but they are overwhelmingly less frequent than low asymmetries, and (*ii*) high asymmetries tend to be concentrated around pairs of very short length between them.

**Figure 3:**
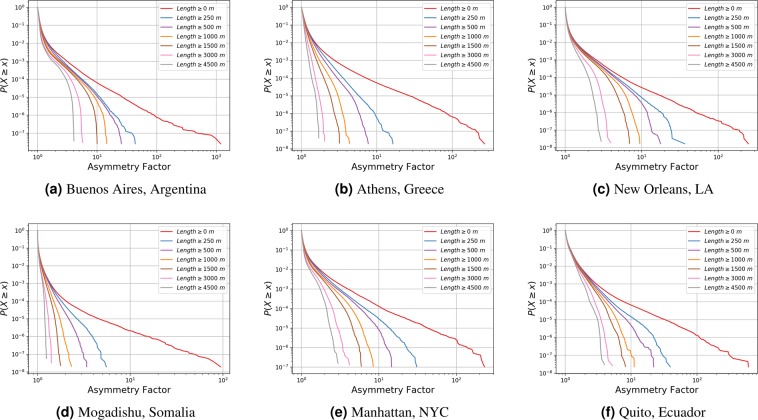
CCDFs on data sets filtered by discarding the asymmetries of all pairs of nodes *u* ≠ *v* ∈ *V* whose distance is below a minimum threshold.

We assess this observation more rigorously in Fig. 3. Let $X\in {{\mathbb{R}}}_{\ge 1}$ be a random variable representing the asymmetry factor of a pair of nodes *u* ≠ *v* ∈ *V* sampled uniformly at random and recall that the Complementary Cumulative Distribution Function (CCDF) is given by *P*(*X* > *x*). First, we filter the data sets by discarding the asymmetries of all pairs of nodes *u* ≠ *v* ∈ *V* whose length between, that is min{*l*(*u*,*v*), *l*(*v*,*u*)}, is below a minimum threshold. Then, in Fig. 3 we plot the respective CCDFs for various values of such threshold. In other words, the curves indicate the probability of observing asymmetries greater than some *x* given that the lengths between all *u* and *v* exceed a minimum threshold. Notice that in all cases, the probability of observing asymmetries greater that some *x* decays by various orders of magnitude with relatively small increments of *x*. The figures also reveal a long tail in the unfiltered data sets (i.e., Length ≥ 0m), which shrinks to the left very quickly for even minuscule filters such as Length ≥ 250m. Moreover, the shrinking of the CCDF exhibits diminishing returns in the minimum length requisite, which is expected since the asymmetries are bounded below by one. These observations not only confirm that high asymmetries are overwhelmingly concentrated around pairs of very short length between them, but they also suggest that the maximum observed asymmetry decreases rapidly towards one as the minimum length threshold increases.

**Figure 4:**
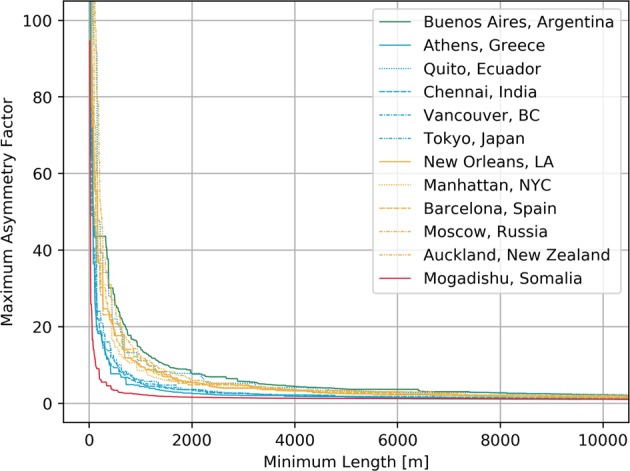
Maximum asymmetry factors observed for all cities under study as a function of the minimum length between any pair of nodes, with a step size of 10 meters. The curves are color-coded by the classification of their corresponding city^19^; green for Category 1, blue for Category 2, orange for Category 3, and red for Category 4.

In Fig. 4 we plot the trajectory of the maximum asymmetry factors given small increments in the minimum length threshold for each of the cities under study. The curves are color-coded according to the topological footprint category of their corresponding city^19^. Note that, barring some possible outliers, the maximum asymmetry factor decay from small to large minimum length filters are qualitatively ranked as follows: Category 4 decays the quickest, followed by Category 2, then by Category 3, and lastly by Category 1. Louf and Barthelemy explain that Category 4 exhibits small square-shaped blocks, Category 2 exhibits small blocks with broadly distributed shapes, Category 3 predominantly exhibits medium-sized blocks, and Category 4 exhibits medium-sized rectangles together with small squares. Thus, one may be tempted to believe that larger city blocks imply a slower asymmetry factor decay, especially when combined with scattered small blocks.

However, while the distribution of block sizes and shapes may indeed influence the asymmetry factors, it fails to fully explain them as it does not consider network topology aspects such as the presence of one way roads. To see the claim, consider a city with two-way roads between every pair of points. Then, all paths are symmetric regardless of the particular distribution of block sizes and shapes. Therefore, the distribution of block sizes and shapes is, for instance, unable to fully explain why the asymmetry factors in Quito, Ecuador decay at a rate similar to that of cities in Category 3, even though the curves of the remainder of the cities in Category 2 are relatively close to each other. We hypothesize that both, the presence of one-way roads and the distribution of block sizes and shapes are both necessary for the generation of high asymmetry factors. To see why the distribution of block sizes and shapes remains important, consider the case in which all blocks are equal-sized squares. Then, even under the presence of one-way roads that circulate around each block, the asymmetry factor will not be very large.

**Figure 5:**
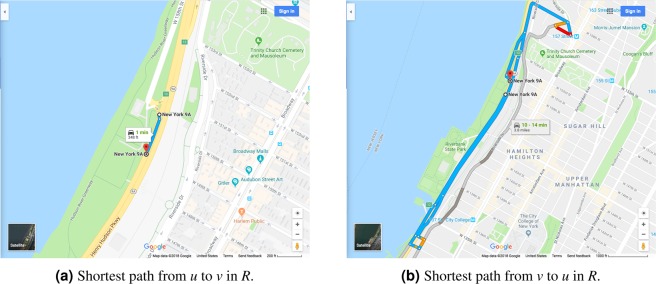
Pair of nodes *u*,*v* with highest asymmetry factor Δ_{*u*,*v*}_ ≈ 214, with min{*l*(*u*,*v*), *l*(*v*,*u*)} ≈ 37 meters, found in the road network *R* of Manhattan, NYC. The nodes *u* and *v* are located at latitude-longitude coordinates (40.8313944, −73.9508067) and (40.8310864, −73.950962). Source: Map data ©2018 Google.

We inspect the proposed generative mechanism for high asymmetries in Fig. 5, where we expose the pair of distinct nodes with the highest asymmetry factor found in Manhattan, NYC. In particular, we observe that the high asymmetry factor between this pair of nodes along the Henry Hudson Parkway is the result of a short segment of road in one direction of the parkway together with the restricted access (e.g., access ramps, prohibited U-turns) and elongated ‘blocks’ (e.g., the median between road directions) induced by this class of roads. Thus, a reasonable generative mechanism is the existence of nodes *u*,*v* with extremely short length between together with the long graph circuits induced by the road restrictions described above. The latter is closely related to the large road network circuities identified by Boeing^20^.

**Figure 6:**
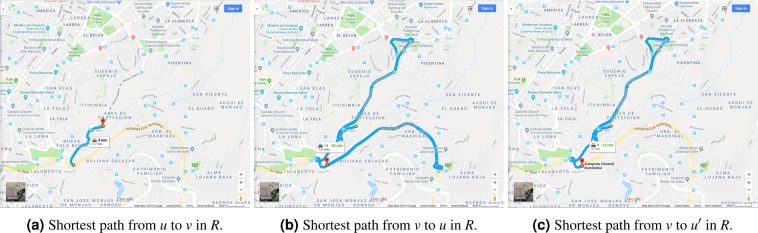
Pair of nodes *u*,*v* with highest asymmetry factor Δ_{*u*,*v*}_ ≈ 11 given a minimum length threshold of *l*(*u*,*v*) ≥ 1,000 meters found in the road network *R* of Quito, Ecuador. The nodes *u* and *v* are located at latitude-longitude coordinates (−0.226874, −78.4977572) and (−0.2333029, −78.502577). The node *u*′ mimics fixing the requirement to visit a node in the ‘wrong side of the road’ by, for instance, walking. Source: Map data ©2019 Google.

We validate the proposed generative mechanism for a minimum length filtered data set in Fig. 6, where we inspect the shortest paths between the pairs of maximum asymmetry given a minimum length threshold of 1,000 meters. Quito, Ecuador is of special interest due to it being an outlier in Category 2 according to Fig. 4. We observe in Fig. 6a,b that, once again, the high asymmetry is due to restricted access roads, in this case a tunnel through a mountainous region together with a restricted access parkway. This is true even in Fig. 6c, where a high asymmetry is observed even after fixing the portion of the path that is an artifact of being in the ‘wrong side of the road’. In this case, the roadway median again induces elongated ‘blocks’. However, anomalies of this kind start disappearing as the minimum length threshold increases, intuitively because the long circulations around the blocks becomes negligible compared to the overall distance travelled. In the case of Quito, the limitations that its mountainous geography inherently impose on road design, and thus on the distribution of block shapes and sizes, may contribute to the slower decay.

## Discussion

The objective of this research is to propose the asymmetry factor Δ_*G*_ of a directed graph *G* as a graph measure with immediate practical implications in discrete optimization. In particular, if we are able to establish that Δ_*G*_ is a small constant, we obtain simple constant factor approximation algorithms for some discrete optimization problems via a symmetrization procedure and the black box use of approximation algorithms for symmetric graphs. Thus, our main contribution is empirically establishing the validity of that premise in the context of road networks.

Our empirical results suggest that the worst-case asymmetry factors become bounded by a small constant when the length between pairs of distinct points in a road network is restricted to be above some possibly small threshold, say 250 meters. This is the main point of Fig. 4, which demonstrates this claim for the twelve cities under study. As seen qualitatively in Figs 5 and 6, the generative mechanism for large asymmetries is the existence of nodes *u*,*v* with extremely short length between together with long circuits induced by restricted access and one-way roads.

It is worth specifying how this observation translates into an algorithmic result. Recall that we began with a graph *R* representing a road network and then prepared a metric closure *G*. We did this because we were interested in analyzing the asymmetry factors for the entire city. In most applications, however, we are not required to do this for the entire set *V*. Consider, for example, a high-capacity ridesharing system^47^ where, in order to drop off the passengers in a vehicle, we need to solve a TSP-like problem only on a subset *V*′ ⊆ *V* of the nodes in *R*, namely those that the passengers set as their origins and destinations. In this application, the origin and destination nodes corresponding to a single requests are likely not too close to each other, as otherwise the user would walk. Now, if distinct requests have origin or destination nodes that are very close too each other, these could potentially by congregated into a single node^48^. In fact, the ridesharing company Uber offers a similar product termed Express Pool. Therefore, one would expect the lengths between the pairs of nodes *u* ≠ *v* ∈ *V*′ to be in the order of a few kilometers, as opposed a few hundred meters. Then, based on our empirical results, we know that the asymmetry factor is a small constant.

It is also worth mentioning that the Δ_*G*_ in the approximation factors obtained is a worst-case performance guarantee. Indeed, from Fig. 3 we know that most pairs are nearly symmetric. Thus, it must be the case that on average, the approximation factors obtained when the algorithm is used are not as high as in the theoretical guarantee. Evaluating this claim on real input instances is an interesting direction of research.

Beyond the algorithmic implications, city planners and engineers may use our methodology to identify and correct sources of chronic large circulations. For example, it may help in finding pairs of points that exhibit frequent travel (e.g., access into and out of a parking garage, taxi depots and passenger hot-spots, shuttles) while also exhibiting a high asymmetry factor. Such pairs would be undesirable, as the vehicles moving in one direction would travel significantly longer than the vehicles traveling the other way around, ultimately increasing the vehicle miles traveled (VMTs), congestion, and pollution. Moreover, city planners and engineers may use our methodology to preemptively identify the effects of transforming certain roads into one-way roads, or vice-versa, so as to pinpoint the potential benefits and drawbacks.

Our results are subject to some limitations. First, many of the applications of interest rely not on the length between two points in a road network, but on the travel time between them. Unfortunately, travel time data may not be collected in certain cities. Moreover, in the cities where such data sets are in fact collected, they may not be publicly available. In the interest of replicability and universality of our research, we opt to treat road length as a reasonable proxy for travel time, especially in the absence of congestion effects. Validating these results with travel time data is an interesting research direction. Second, the crowd-sourced nature of Open Street Map may present a challenge in the quality of the data in underrepresented or conflicted parts of the world.

## Supplementary information

Supplementary Material

## Data Availability

The source code developed to obtain the results presented will be provided through a publicly available repository. The libraries and data sets^25–29^ used within the source code are publicly available.
