# Supplementary material for: Bounded Asymmetry in Road Networks
Source: Sci Rep. 2019 Aug 16;9:11951. doi: 10.1038/s41598-019-48463-z (PMC6697698; doi:10.1038/s41598-019-48463-z)
Supplement: Supplementary file 1 — Supplementary Material [file 41598_2019_48463_MOESM1_ESM.pdf]

Bounded Asymmetry in Road Networks

Juan C. Martínez Mori[1,*] and Samitha Samaranayake[2]

[1]Center for Applied Mathematics, Cornell University, Ithaca, NY, 14853, USA
[2]School of Civil and Environmental Engineering, Cornell University, Ithaca, NY, 14853, USA
[*]jm2638@cornell.edu

Supplementary Material

Table S1 summarizes the bounding box coordinates, namely North and South (latitude) and East and West (longitude) for each of the cities studied. The bounding boxes are selected by hand via Open Street Map's user interface [26]. The intent is to capture the topology of the network near each city's central core while maintaining a tractable network size.

	Bounding Box Coordinates			
City	North	South	East	West
Buenos Aires, Argentina	−34.5583	−34.6519	−58.3504	−58.4819
Athens, Greece	38.0125	37.9507	23.7653	23.6932
Quito, Ecuador	−0.1428	−0.2547	−78.4297	−78.5368
Chennai, India	13.1263	13.0370	80.3015	80.2201
Vancouver, BC	49.3024	49.2147	−123.0283	−123.2666
Tokyo, Japan	35.7047	35.6609	139.8000	139.7277
New Orleans, LA	30.0379	29.9097	−90.0422	−90.1600
Manhattan, NYC	N/A	N/A	N/A	N/A
Barcelona, Spain	41.4185	41.3281	2.2343	2.1104
Moscow, Russia	55.8183	55.6950	37.7452	37.5032
Auckland, NZ	−36.8030	−36.9123	174.8508	174.6878
Mogadishu, Somalia	2.0660	2.0107	45.3699	45.2995

Table S1. Bounding box coordinates of each of the cities studied, which we query through `OSMNX` [25, 26]. The bounding box for Manhattan, NYC is unavailable since we query this network *by place*, i.e., instead of a bounding box, the query uses the string 'Manhattan, NYC'. This query returns the network in Figure 1a

Figure S1 presents scatter plots of the asymmetry factors found in the eight remaining cities not presented in the Results section. Similarly, Figure S2 presents the respective CCDF curves. The observations made in the Results section regarding the scatter and CCDF plots remain unchanged for all cases. Moreover, the maximum asymmetry factors observed for different values of the minimum length threshold in Figure S2 are part of the trajectories discussed earlier in Figure 4.

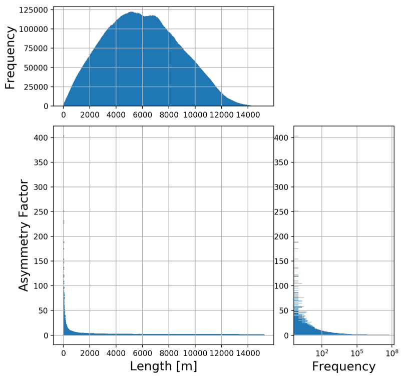

(a) Chennai, India

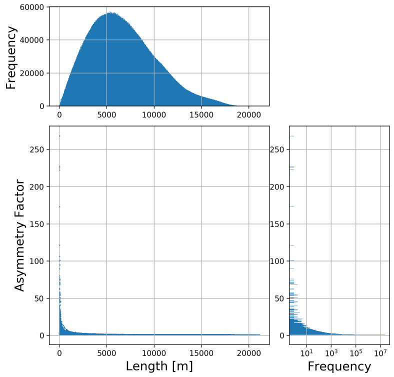

(b) Vancouver, BC

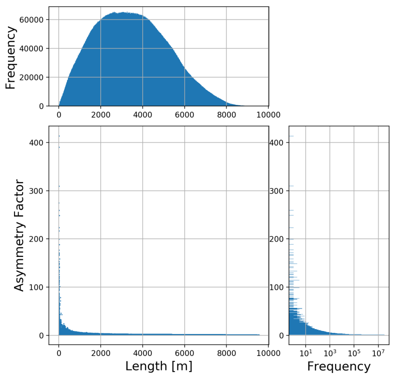

(c) Tokyo, Japan

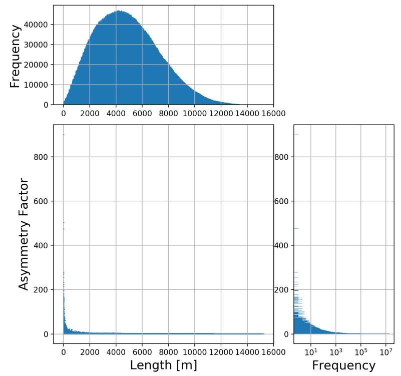

(d) Barcelona, Spain

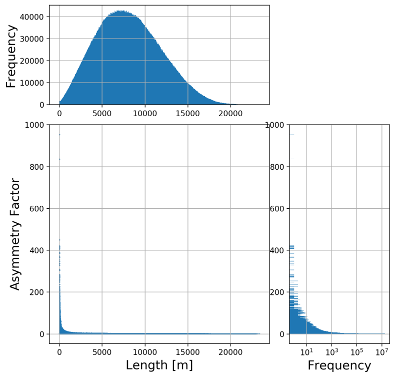

(e) Moscow, Russia

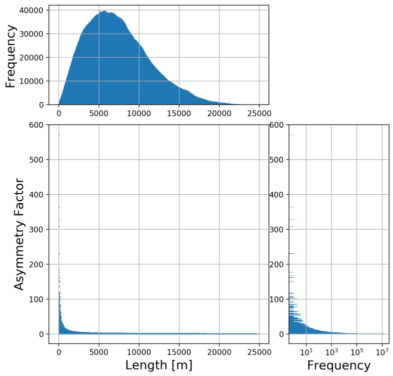

(f) Auckland, NZ

Figure S1. Supplement of Figure 2.

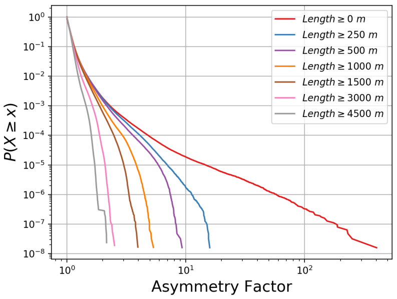

(a) Chennai, India

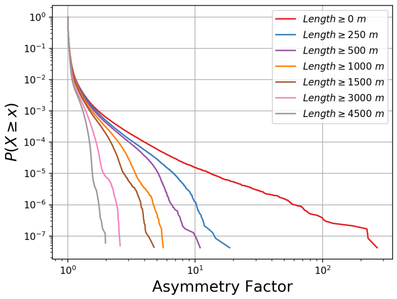

(b) Vancouver, BC

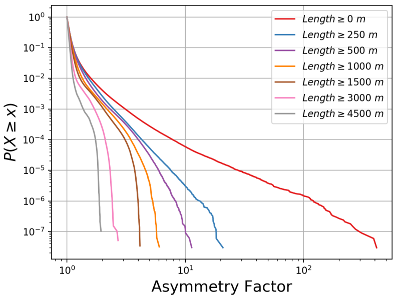

(c) Tokyo, Japan

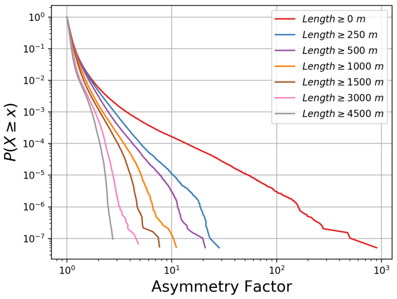

(d) Barcelona, Spain

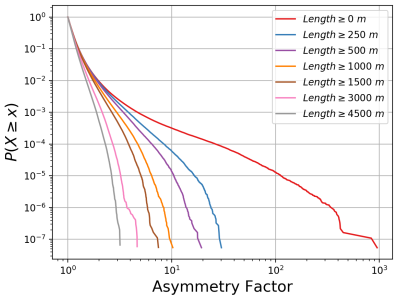

(e) Moscow, Russia

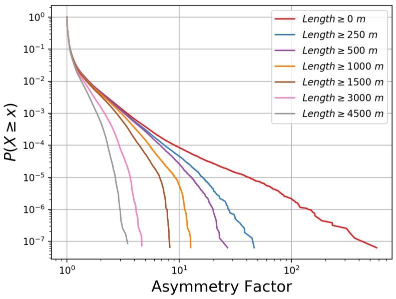

(f) Auckland, NZ

Figure S2. Supplement of Figure 3.
